# Development of acoustically isolated extracellular plasma vesicles for biomarker discovery in allogeneic hematopoietic stem cell transplantation

**DOI:** 10.1186/s40364-020-00259-4

**Published:** 2021-01-19

**Authors:** Hooi Ching Lim, Shamit Soneji, Róbert Pálmason, Stig Lenhoff, Thomas Laurell, Stefan Scheding

**Affiliations:** 1 Division of Molecular Hematology, Department of Laboratory Medicine, Lund Stem Cell Center, Lund University, BMC B12, Klinikgatan 26, 22184 Lund, Sweden; 2 Department of Hematology, Skåne University Hospital, Lund, Sweden; 3 Division of Nanobiotechnology and Lab-on-a-chip, Department of Biomedical Engineering, Lund University, Lund, Sweden

**Keywords:** Acoustic trapping, Plasma extracellular vesicles, EVs, Allogeneic hematopoietic stem cell transplantation, Allo-HSCT, Transplantation, Biomarker development, Infection, Graft-versus host disease, GvHD

## Abstract

**Background:**

Infection and graft-versus-host disease (GvHD) are the major causes for mortality and morbidity of allogeneic hematopoietic stem cell transplantation (allo-HSCT). Plasma-derived extracellular vesicles (EVs) contain disease-related proteins, DNAs and RNAs, and have recently been suggested as potential biomarker candidates for transplantation complications. However, EV isolation from small plasma volumes in clinical biomarker studies using conventional methods is challenging. We therefore investigated if EVs isolated by novel automated acoustic trapping could be developed as potential biomarkers for allo-HSCT complications by performing a clinical proof-of-principle study.

**Results:**

Plasma samples were collected from twenty consecutive patients with high-risk/relapsed hematologic malignancies undergoing allo-HSCT before transplantation and post-transplant up to 12 weeks. EVs were isolated from small plasma sample volumes (150 μl) by an automated, acoustofluidic-based particle trapping device, which utilizes a local λ/2 ultrasonic standing wave in a borosilicate glass capillary to capture plasma EVs among pre-seeded polystyrene microbeads through sound scatter interactions. We found that EVs could be reliably isolated from all plasma samples (*n* = 173) and that EV numbers increased more than 2-fold in the majority of patients after transplantation. Also, sufficient quantities of RNA for downstream microRNA (miRNA) analysis were obtained from all samples and EV miRNA profiles were found to differ from whole plasma profiles. As a proof of principle, expression of platelet-specific miR-142-3p in EVs was shown to correlate with platelet count kinetics after transplantation as expected. Importantly, we identified plasma EV miRNAs that were consistently positively correlated with infection and GvHD, respectively, as well as miRNAs that were consistently negatively correlated with these complications.

**Conclusions:**

This study demonstrates that acoustic enrichment of EVs in a clinical biomarker study setting is feasible and that downstream analysis of acoustically-enriched EVs presents a promising tool for biomarker development in allo-HSCT. Certainly, these findings warrant further exploration in larger studies, which will have significant implications not only for biomarker studies in transplantation but also for the broad field of EV-based biomarker discovery.

**Supplementary Information:**

The online version contains supplementary material available at 10.1186/s40364-020-00259-4.

## Background

Complications after allogeneic hematopoietic stem cell transplantation (allo-HSCT) such as infection and graft-versus-host disease (GvHD) can be severe and cause substantial mortality and morbidity. Early diagnosis of transplant-related complications is critical to allow timely initiation of effective treatments. However, diagnosis especially of GvHD can be difficult. A number of proteins, such as TNFR-1, IL-2Rα, Reg3α and ST2 have been validated as GvHD biomarkers and recently reported algorithms using marker combinations allow for the prediction of early treatment response [1–3]. However, thus far none of the reported methods reliably predicts the occurrence of GvHD, which would allow to promptly initiate early and effective therapeutic interventions.

On the other hand, small non-coding microRNAs (miRNAs) isolated from plasma have been suggested as potential predictive acute GvHD (aGvHD) biomarkers that correlated with disease severity and survival, but studies are still in their early stages [4, 5]. In addition to free miRNAs in plasma, miRNAs can be packaged into small phospholipid membrane enclosed vesicles, so-called extracellular vesicles (EVs) [6]. Importantly, the molecular composition of EVs is reflective of the changes that occur in the cells of their origin and, accordingly, molecular profiling of EVs has a considerable potential as prognostic biomarkers for pathological conditions, such as GvHD.

EVs are conventionally isolated by ultracentrifugation, which is the gold standard. However, ultracentrifugation is laborious, time consuming, requires large sample volumes, and produces inconsistent results across different laboratories due to the differences in protocols [7–9]. Our study therefore aimed to investigate if miRNA expression profiling in plasma extracellular vesicles collected by acoustic trapping, a novel ultrasound-based EV isolation method, could be used for biomarker development in patients undergoing allo-HSCT. In contrast to ultracentrifugation and other EV isolation methods [10, 11], acoustic trapping is an automated and label-free microfluidic technology which enables enrichment of EVs from small sample volumes with minimal sample preparation [12–14].

To develop a broadly applicable technology that potentially can be translated to analyse small plasma samples in large cohort studies, we herein demonstrate in a pilot clinical study with 20 allo-HSCT patients that acoustic trapping allowed to enrich sufficient numbers of EVs from plasma for nanoparticle tracking analysis and miRNA panel profiling. Furthermore, our data showed that acoustically enriched plasma EVs correlated with aGvHD and infection, and thus represent promising non-invasive biomarkers for the diagnosis of clinical complications after allo-HSCT. More importantly, this real-life study provides proof-of-principle evidence that acoustically-enriched EVs can be utilized for biomarker studies on large cohorts of patients in a broad variety of diseases.

## Methods

### Patient characteristics and plasma sampling

The study cohort comprised of 20 consecutive patients (age: 21–71 years) with high-risk or refractory/relapsed diseases who underwent allo-HSCT at Skåne University Hospital, Lund, Sweden. The patients’ characteristics are provided in Table 1. Transplantations were performed with mobilized peripheral blood progenitor cells from related (*n* = 5) and unrelated donors (*n* = 15) after standard conditioning regimen. All patients received cyclosporine and methotrexate as GvHD prophylaxis. Whole blood samples were collected in sodium citrate vacutainers (Becton Dickinson, New Jersey, USA) before conditioning and HSC transplantation (week 0), weekly post-HSCT until discharge from the ward and bi-weekly thereafter until week 12. Platelet free plasma was obtained by two serial centrifugations at 1600×g for 15 min at room temperature. Plasma samples were aliquoted and kept frozen at − 80 °C. Clinical and laboratory data were collected by chart review.

**Table 1:** Patients characteristics

	Age/gender (m/f)	Disease/− stage	Stem cell source, HLA matching	Donor type/age/ gender	Conditioning regimen	CMV, EBV status (R/D)
Pat 1	51/m	MM/relapse after autologous Tx	PB, 11/12	MUD/22/ m	Flu/Tre/ATG	+/−, +/+
Pat 2	37/m	Sezary’s syndrome	PB, full	MRD/47/m	Cy/TBI	+/−, +/+
Pat 3	35/f	hrAML	PB, full	MUD/20/f	Flu/Bu4/ATG	+/+, +, n.d.
Pat 4	46/m	hrAML	PB, 8/8	MUD/29/f	Flu/Bu4/ATG	+/−, +/+
Pat 5	21/m	preB-ALL, CR2	PB, 7/8	MUD/43/m	Cy/TBI/ATG	+/−, +/+
Pat 6	63/m	MDS, RAEB 1	PB, full	MRD/59/m	FluTre	−/−, +/+
Pat 7	68/m	AML, IR	PB, 8/8	MUD/34/m	Flu/Bu2/ATG	+/+, −/n.d.
Pat 8	59/f	Post-ET myelofibrosis	PB, 11/12	MUD/23/m	Flu/Bu3/ATG	+/+, +/+
Pat 9	27/f	hrMDS	PB, full	MUD/38/m	Flu/Tre/ATG	+/−, +, n.d.
Pat 10	69/m	MDS/AML	PB, 10/12	MUD/37/m	FluBu2ATG	+/−, +/+
Pat 11	64/m	MDS/AML	PB, 10/12	MUD/26/f	Flu/Tre/ATG	+/−, +/n.d.
Pat 12	71/m	hrMDS	PB, 10/12	MUD/35/m	Flu/Tre/ATG	+/−, +/n.d.
Pat 13	60/m	MDS/AML	PB, full	MRD/57/m	FluTre	−/−, +/+
Pat 14	67/f	Post-ET myelofibrosis	PB, 10/12	MUD/30/f	Flu/Bu3/ATG	−/+, +/+
Pat 15	64/f	hrAML	PB, 11/12	MUD/20/f	Flu/Bu3/ATG	+/+, +/+
Pat 16	66/m	MCL, relapse after autologous Tx	PB, 10/12	MUD/34/m	Flu/Cy/TBI/ATG	+/−, +/n.d.
Pat 17	42/m	MDS/AML	PB, full	MRD/56/f	FluTre	+/+, +/+
Pat 18	21/f	AML, CR2	PB, full	MRD/19/m	Flu/Bu4	+/+, n.d./n.d.
Pat 19	67/m	hrAML	PB, 11/12	MUD/24/m	Flu/Bu2/ATG	+/−, +/n.d.
Pat 20	30/m	hrT-ALL	PB/ full	MRD/33/m	Cy/TBI	+/+, +/+

Abbreviations: *m* male, *f* female, *HLA* human leukocyte antigen, *MUD* matched unrelated donor, *MRD* matched related donor, *CMV* cytomegalovirus, *EBV* Ebstein Barr virus, *D* donor, *R* recipient, *PB* peripheral blood stem cells, *Tx* transplantation, *n.d.* not determined, *hrAML* high-risk acute myeloid leukemia, *preB-ALL* acute pre-B lymphoblastic leukemia, *MDS* myelodysplastic syndrome, *RAEB1* refractory anemia with excess blasts 1, *IR* intermediate risk, *CR2* second complete remission, *ET* essential thrombocytosis, *hrT-ALL* high-risk acute T-lymphoblastic leukemia, *MCL* mantle cell lymphoma

Conditioning regimens: Cy/TBI: cyclophosphamide 120 mg/kg, fractionated total body irradiation12 Gy Cy/TBI/ATG: cyclophosphamide 120 mg/kg, fractionated total body irradiation 12 Gy, antithymocyte globulin 6 mg/kg

Flu/Cy/TBI/ATG: fludarabine 180 mg/m^2^, cyclophosphamide 60 mg/kg, fractionated total body irradiation 6 Gy, antithymocyte globulin 6 mg/kg

Flu/Tre: fludarabine 150 mg/m^2^, treosulfan 2.8 g/m^2^ Flu/Tre/ATG: fludarabine 150 mg/m^2^, treosulfan 2.8 g/m^2^, antithymocyte globulin 4 mg/kg Flu/Bu2, (3), (4)/ATG: fludarabine 150 mg/m^2^, busulfan 6.4 mg/kg, (9.6 mg/kg), (12.8 mg/kg); antithymocyte globulin 4 mg/kg

### Extracellular vesicle enrichment by acoustic trapping

**Fig. 1:**
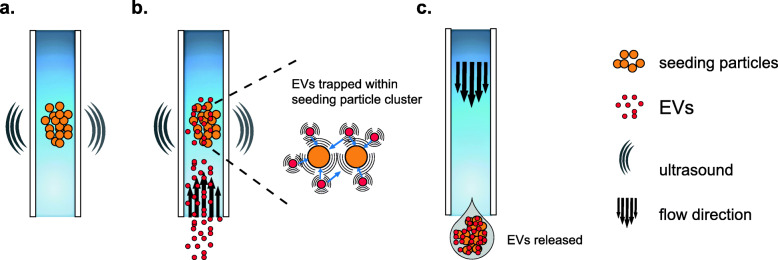
Schematic illustration of acoustic trapping with seeding particles for EV enrichment. **a** Seeding particles are aspirated and trapped in the acoustic standing wave field generated by a piezoelectric transducer. **b** Plasma containing EVs is aspirated (at 15 μL/min) and EVs are trapped together with the seeding particle cluster by the acoustic forces induced by scattered sound particle-particle interaction. **c** The trapped EV and seeding particle cluster is washed and subsequently released for further analysis after the acoustic field is turned off

Acoustic trapping was performed using the AcouTrap instrument (AcouSort AB, Lund, Sweden) as previously described [12, 13] (Fig. 1). The experimental parameters applied such as flow rate, voltage and seeding particle size were determined in our previous optimisation studies [12, 15] and preceding optimization experiments using polystyrene beads. These settings provided for sufficiently high EV isolation using the version of the AcouTrap machine that was employed in our study. Acoustic trapping utilises a local ultrasonic λ/2 acoustic standing wave produced by a 1 mm wide piezoelectric transducer glued to a borosilicate glass capillary and operated at approximately 4 MHz, 9.5 V peak-to-peak sinusoidal wave. Briefly, 12 μm polystyrene beads (Sigma-Aldrich, Missouri, USA) serving as seeding particles were trapped and washed with 50 μL of Phosphate Buffered Saline (PBS) followed by aspiration of diluted plasma (1:2 dilution with PBS) at 15 μL/min at room temperature. EVs from the biological fluids are acoustically captured among polystyrene beads pre-seeded in the trap by particle/particle interaction in the scattered acoustic field, and are then released for downstream analysis. Fifty microliter and three hundred microliter of diluted plasma (1:2 with PBS) were processed to collect EVs for nanoparticle tracking analysis and miRNA profiling, respectively. After sample aspiration and EV enrichment, the EV-containing seed particle cluster was washed in the trap with PBS before releasing the cluster by turning off the transducer and eluting the sample in PBS.

### Extracellular vesicle number and size measurements

The number and size distribution of EVs were analysed with a NanoSight LM10 equipped with a continuous syringe pump system (Malvern Inc., Malvern, UK). The screen gain was set to 1 and camera level was set to 10 in the acquisition software (NTA version 3.2). All samples were measured 3 times for 30 s each with a detection threshold of 10.

### Transmission electron microscopy

For transmission electron microscopy (TEM), enriched EV samples were prepared using the protocol described previously [16]. Specimens were examined with a Tecnai Spirit BioTWIN transmission electron microscope (TEI, Oregon, USA).

### RNA isolation

Total RNA was isolated from 150 μL of whole plasma and acoustically enriched EVs from the same amount of plasma in 500 μL buffer using the miRNeasy Serum/Plasma Kit (Qiagen, Hilden, Germany) following the manufacturer’s instructions. The elution volume for both sample types was 25 μL in RNase-free water.

### MicroRNA profiling assay using real-time quantitative polymerase chain reaction (qPCR)

MicroRNA profiling was performed by Qiagen Genomic Services (Qiagen, Hilden, Germany). Briefly, RNA (7 μL) was reverse transcribed using the miRCURY LNA RT Kit (Qiagen, Hilden, Germany). cDNA was diluted 50× and assayed in 10 μL PCR reactions according to the protocol for miRCURY LNA miRNA PCR; each miRNA was assayed once by qPCR on the miRNA Ready-to-Use PCR, Serum/Plasma Focus panel consisting of 179 miRNAs using miRCURY LNA SYBR Green master mix. Negative controls excluding template from the reverse transcription reaction were performed and profiled like the samples. The amplification was performed using a LightCycler® 480 Real-Time PCR System (Roche, Mannheim, Germany) in 384 well plates. The amplification curves were analysed using the Roche LC software.

### Data analysis

All qPCR data were normalized to the average of assays detected in all samples or alternatively to the average of custom defined assays detected in all samples [17, 18]. For the correlation analysis, each miRNA was correlated to GvHD and infection for individual patients using Spearman rank correlation. The infection correlation analysis was performed on EV profiles from patients fulfilling the criteria for infection in weeks 1 to 6 (Table 2). The GvHD correlation analysis was performed on patients who were diagnosed with GvHD at ≥3 time points. For both analyses, the correlation of each miRNA to a patient was split according to positive/negative correlation to the GvHD/infection profiles. These miRNAs were then ranked for consistency by sorting on the z-score of correlation coefficients across patients. The heatmap of expression profiles on EV and corresponding whole plasma was generated by using unsupervised hierarchical clustering of miRNAs in EVs and plasma, respectively, based on the top 25 miRNAs with highest standard deviation. The heatmap was generated using the pheatmap library for R using default parameters.

**Table 2:** Post-transplant complications

	# affected patients (pat-ID)								
Complication^**a**^	Pre/Tx	week 1	week 2	week 3	week 4	week 6	week 8	week 10/11	week12
Early infection, neutropenic (week 1–4)	4^b^ (3, 10, 11, 19)	7 (1,2,5, 7,11,12,19)	11 (2,5,6,8,9,11,12,13,16,19,20)	5 (5,9,12,14,16)	2 (5,16)				
Late Infection (≥ week 5)								2 (1,8)	1 (1)
GvHD (all grades)			1	1	2	7	7	7	5
Grade I			1 (2)	1 (2)	2 (1,8)	7 (1,2,4,7,8,9,17)	5 (1,2,6,7,17)	5 (2,4,5,6,7)	4 (2,4,10,18)
Grade II/III							2 (8,19)	2 (8,19)	1 (8)
CMV reactivation						3 (1,4,16)	2 (16,19)	1 (19)	1 (8)

^a^Definition of complications: Infection (use of broad spectrum i.v. antibiotics because of fever > 38.5 °C plus CRP > ULN [upper limit of normal]); GvHD (overall grades according to guidelines from Mount Sinai Acute GvHD International Consortium; CMV reactivation (detectable copies of CMV DNA in the peripheral blood plus CMV-active antiviral treatment)

^b^ neutropenic fever on day of transplantation due to antithymocyte globulin (ATG)

## Results

### Patients’ clinical course and complications

Twenty patients underwent conditioning treatment and allogeneic hematopoietic stem cell transplantation (allo-HSCT). Complications related to conditioning and transplantation included fever (> 38.5 °C) and elevated c-reactive protein (CRP) levels due to antithymocyte globulin treatment observed in four patients (Table 2). Post-transplantation neutropenic fever requiring broad-spectrum antibiotics was recorded in 14 patients (Table 2) and two patients developed infectious complications later in the course with neutrophils < 0.5 × 10^9^ /L (patient 1) and > 1 × 10^9^ /L (patient 8), respectively. A total of 13 patients developed GvHD at a median onset of 6 weeks after transplantation (range: 2–12). Eleven patients had GvHD grade I which exclusively involved the skin, one patient (patient 19) had GvHD grade II and one patient (patient 8) developed grade III GvHD with skin, liver and gut involvement (Table 2). GvHD treatment was initiated in three patients with corticosteroids only; the patient with grade III GvHD received several lines of therapy and eventually died of GvHD-related complications 5 months after HSCT. One patient with early post-transplant relapse of his Ph + pre-B-ALL 10 weeks post-transplant (patient 5) underwent additional chemotherapy and finally died of leukemia 5 months after HSCT. All patients were alive at study end, i.e. 3 months post-transplantation.

### Numbers and basic properties of acoustically-isolated plasma EVs in Allo-HSCT patients

**Fig. 2:**
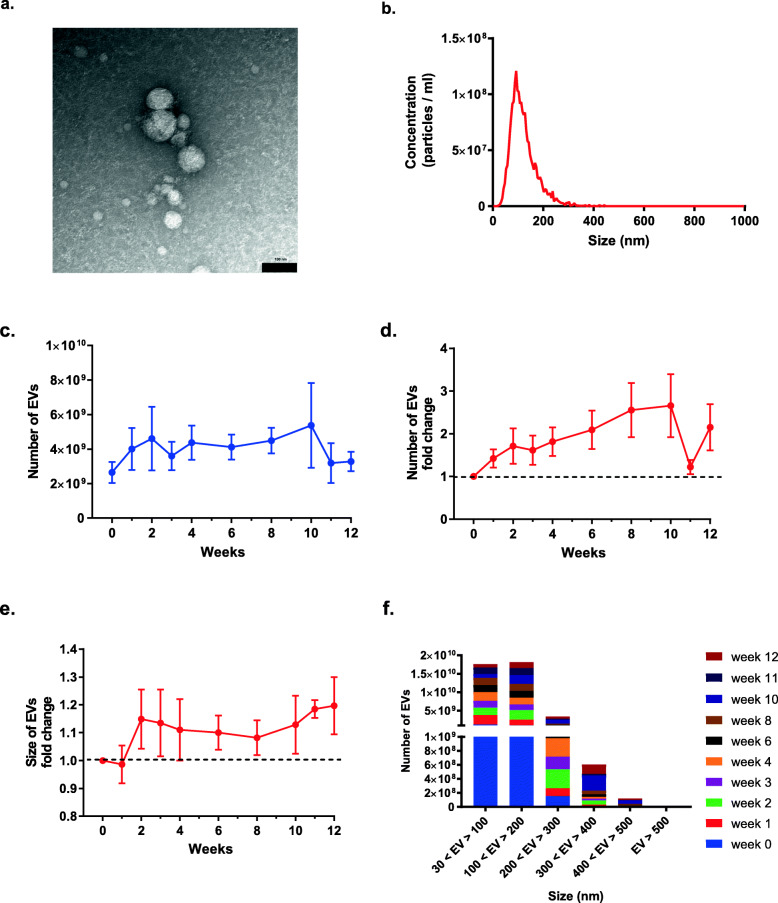
Increased numbers of EVs are released into the plasma after allo-HSCT. **a** Transmission electron image of acoustically enriched EVs, bar = 100 nm. **b** Size distribution of EVs as measured by nanoparticle tracking analysis (NTA). Absolute numbers of EVs (**c**) and fold change of EVs (**d**) (per 50 μL diluted plasma) increased after allo-HSCT compared to pre-transplantation values. Pre-transplantation = week 0; weeks after transplantation = week 1, 2, 3, 4, 6, 8, 10, 11, 12. **e** After transplantation, EV sizes slightly increased over time compared to week 0. **f** Size distribution of EVs at different time points before and after transplantation. EV numbers are indicated per 50 μL diluted plasma. Data in b and c are presented as mean ± SEM of 10 patients

EVs were enriched from patient plasma samples collected before and at sequential time points after transplantation using acoustic trapping. Acoustically enriched EVs were intact, round and heterogeneous in size which is consistent with the literature [19] (Fig. 2a and b). Median numbers of EVs recovered from 50 μL diluted plasma (1:2) were 1.9 × 10^9^ (range 3.7 × 10^8^ - 5.5 × 10^9^) at pre-transplant compared to 2.9 × 10^9^ (range 4.4 × 10^8^–1.5 × 10^10^) after transplantation (Fig. 2c). Interestingly, the majority of patients had increased numbers of EVs in the plasma over time (2–7 fold) (Fig. 2d), indicating that conditioning regimen and post-transplantation conditions promoted the release of EVs, which is consistent with previous studies [20, 21]. In addition, EV size was slightly increased after transplantation (Fig. 2e). Of note, the acoustically enriched EVs were predominately 30–200 nm in size (Fig. 2f).

### MicroRNA expression in EVs is distinct from whole plasma miRNA profiles

**Fig. 3:**
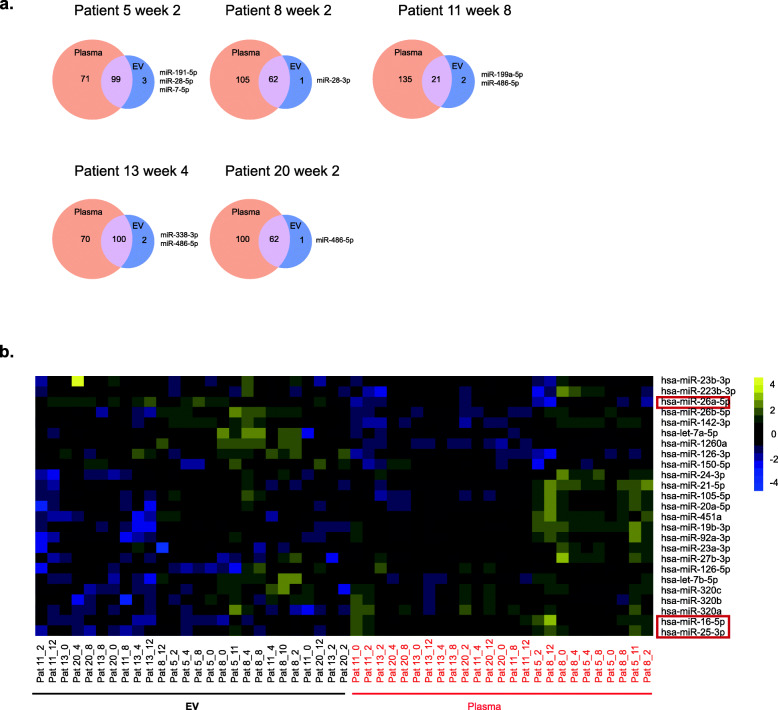
MicroRNA profiling demonstrated distinct miRNA expression profiles in EVs and corresponding whole plasma samples. **a** Venn diagrams show the differential expression of miRNAs in EV and plasma samples (*n* = 5). **b** The heat map shows the unsupervised hierarchical clustering of miRNAs in EVs and plasma samples based on the top 25 miRNAs with highest standard deviation. Red squares mark the representative miRNAs which were differently expressed in EVs and plasma

We performed miRNA profiling of EVs and corresponding whole plasma samples in 5 patients at 5 time points each. Not all EV miRNAs were detected in the corresponding plasma sample (Fig. 3a), indicating that miRNAs were more stable when encapsulated in EVs [22]. Moreover, expression levels of a number of miRNAs were different between EVs and whole plasma samples as shown in unsupervised hierarchical clustering analysis (Fig. 3b). For example, expression of miR-26a-5p was generally higher in EVs, while miR-16-5p and miR-25-3p expression was lower in EVs. In addition, all EV samples were clustered together and separated from whole plasma samples, pointing to biological differences between the different sample types (Fig. 3b). Taken together, these data clearly demonstrate that miRNA profiles for EVs differ from the cell free miRNA profiles in whole plasma and that miRNAs are differentially packaged into EVs in a HSCT setting.

### MicroRNA expression in acoustically enriched EVs from Allo-HSCT patients

Twenty-two EV miRNAs were differently expressed when comparing pre-transplantation (week 0) with week 1 samples, but differences were not statistically significant (Table 3). Some let-7 family miRNAs (let-7e-5p, let-7b-5p, let-7 g-5p, let-7d-3p and let-7d-5p) were downregulated at week 1, suggesting that deregulation of these miRNAs might be due to the conditioning regimen as shown previously [23].

**Table 3:** Top most differentially expressed miRNAs, showing fold change between the week 0 and week 1

miR name	Fold change	***p***-value	BH adj. ***p***-value
hsa-let-7e-5p	−2.9	0.0011	0.064
hsa-miR-7-5p	−2.3	0.0018	0.064
hsa-miR-328-3p	−2.3	0.0024	0.064
hsa-let-7 g-5p	−2.1	0.0028	0.064
hsa-let-7b-5p	−2.1	0.0042	0.064
hsa-miR-16-5p	−1.7	0.0046	0.064
hsa-miR-106b-5p	−2.0	0.0049	0.064
hsa-miR-140-3p	−2.5	0.0058	0.066
hsa-miR-451a	−2.5	0.0068	0.066
hsa-miR-320d	−6.1	0.0073	0.066
hsa-miR-320a	−1.8	0.0088	0.073
hsa-miR-335-3p	−3.4	0.0112	0.085
hsa-miR-30e-5p	−2.1	0.0212	0.138
hsa-miR-205-5p	−3.0	0.0267	0.162
hsa-let-7d-3p	−4.4	0.0321	0.166
hsa-let-7d-5p	−2.3	0.0326	0.166
hsa-miR-30a-5p	−3.3	0.0334	0.166
hsa-miR-18b-5p	−2.5	0.0350	0.166
hsa-miR-93-5p	−1.7	0.0364	0.166
hsa-miR-320b	−2.2	0.0397	0.172
hsa-let-7b-3p	−3.1	0.0422	0.175

The last two columns show the *p*-value from the t-test and the Benjamini-Hochberg (BH) adjusted *P*-value

**Fig. 4:**
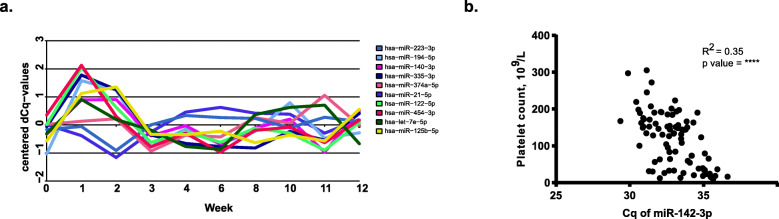
MicroRNA expression in acoustically enriched EVs from allo-HSCT patients. **a** Relative change of the top 10 differently expressed miRNAs over time identified by using Anova analysis (*n* = 20). **b** Expression of miR-142-3p in EVs significantly correlated with peripheral blood platelet counts. Pearson coefficient correlation, *r* = 0.35, *p* = **** (*p* < 0.0001)

Comparison of the miRNA expression at all time points using one-way ANOVA showed that 19 miRNAs were differently expressed and that changes of four miRNAs (miR-223-3p, miR-194-5p, miR-140-3p, miR-335-3p) were statistically significant (Table 4). Interestingly, all of these miRNAs have been reported in the context of haematological malignancies, e.g. acute myeloid leukaemia [24–27], which is consistent with the disease background of the patients (Table 1). Furthermore, miRNA expression patterns were changing across different time points (Fig. 4a), suggesting that EV encapsulated miRNAs could possibly be part of the pathophysiological processes of allo-HSCT complications.

**Table 4:** Top most differentially expressed miRNAs across time points

miR name	***p***-value	BH adj. ***p***-value
hsa-miR-223-3p	5.8e-08	0.0000055
hsa-miR-194-5p	0.0003	0.016
hsa-miR-140-3p	0.001	0.045
hsa-miR-335-3p	0.002	0.048
hsa-miR-374a-5p	0.003	0.065
hsa-miR-21-5p	0.004	0.074
hsa-miR-122-5p	0.006	0.087
hsa-miR-454-3p	0.009	0.111
hsa-let-7e-5p	0.010	0.111
hsa-miR-125b-5p	0.013	0.121
hsa-miR-205-5p	0.014	0.121
hsa-miR-193a-5p	0.015	0.121
hsa-miR-148a-3p	0.017	0.125
hsa-miR-10b-5p	0.020	0.133
hsa-miR-150-5p	0.023	0.143
hsa-miR-30c-5p	0.033	0.194
hsa-miR-484	0.037	0.194
hsa-miR-320d	0.046	0.230

The last two columns show the *p*-value from the ANOVA and the Benjamini-Hochberg (BH) adjusted *p*-value

### Correlation of EV miRNA with peripheral blood platelet counts

As a proof of principle and to demonstrate that acoustically enriched EVs contain specific miRNAs that reflected a certain (patho) physiological condition, we examined the expression of the known platelet-released miRNA miR-142-3p in acoustically enriched EVs in relation to peripheral blood platelet counts. As shown in Fig. 4b and as predicted, expression of miR-142-3p was positively correlated with the platelet counts, which is in concordance with a previous study [28]. Hence, these data confirm that acoustic trapping can be used to enrich EVs that reflect dynamic platelet count changes and likely also other conditions.

### EV miRNA expression patterns correlated with post-transplant infections and GvHD

**Fig. 5:**
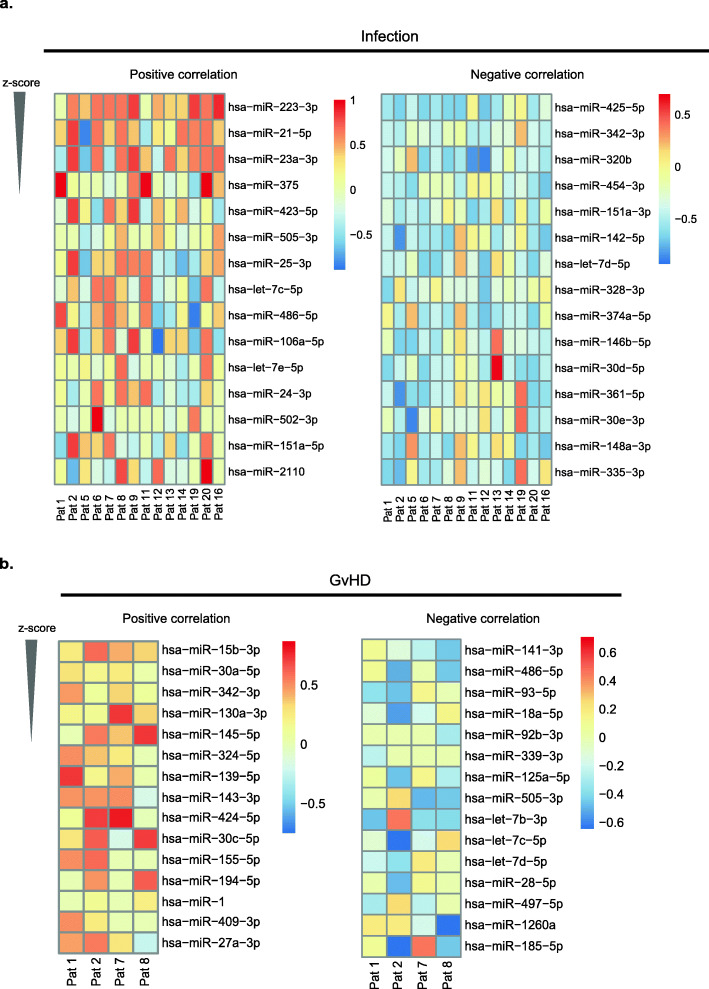
Spearman rank correlation analysis identified miRNAs that consistently positively and negatively correlated with infectious complication and GvHD. Analysis for the top 15 miRNAs that positively and negatively, respectively, correlated with (**a**) infectious complication (*n* = 14) and (**b**) GvHD (*n* = 4). MiRNAs are ranked according to their z-scores and the correlation coefficient values are indicated by the colour scale

Motivated by the correlation of EV miRNAs and platelet counts, we investigated the potential of miRNA candidates as non-invasive biomarkers for clinical complications, i.e. infection and GvHD. We performed Spearman rank correlation analysis for patients with infections in weeks 1 to week 6 since the majority of patients showed early infection episodes after transplantation. Interestingly, EV miRNA analysis identified miR-223-3p, miR-21-5p, miR-23a-3p, miR-375 and miR-423-5p as the top five miRNAs that consistently positively correlated with infectious episodes. On the other hand, miR-425-5p, miR-342-3p, miR-320b, miR-454-3p and miR-151a-3p were the top five miRNAs that consistently negatively correlated with this complication (Fig. 5a, Supplementary Table 1).

Regarding GvHD, our correlation analysis revealed that expression of miRNAs miR-15b-3p, miR-30a-5p, miR-342-3p, miR-130a-3p and miR-145-5p were among the top five that were positively correlated with GvHD (Fig. 5b). Furthermore, miRNAs miR-141-3p, miR-486-5p, miR-93-5p, miR-18a-5p and miR-92b-3p were negatively correlated with GvHD status (Fig. 5b, Supplementary Table 2).

## Discussion

Clinical complications such as infection and, importantly, GvHD remain the most challenging risk factors in allogeneic stem cell transplantation. Thus, a non-invasive diagnostic test to predict the occurrence of complications is needed to enable timely pre-emptive therapy and ultimately reduce morbidity and mortality.

Our study demonstrated that EVs enriched by acoustic trapping could potentially provide new and exciting prospects for the development of non-invasive biomarkers for post-transplant clinical complications. We herein demonstrate that sufficient amounts of plasma EVs could be enriched by acoustic trapping from as little as 150 μL of whole plasma to successfully perform downstream miRNA panel profiling, which extents prior findings that acoustically-enriched plasma EVs can be used for protein profiling [29]. Thus, this automated trapping technology holds great potential to be translated into routine clinical use to enrich EVs for dynamic complication and disease monitoring. Furthermore, it provides a likely superior alternative way to facilitate the process of biomarker discovery on large sample numbers compared to standard EV isolation by ultracentrifugation, which still is the gold standard in the field, as well as other methods, such as overnight precipitation, chromatography and bead isolation [10, 11]. Of note, acoustic trapping technology has been further developed in the course of this study, including technical modifications such as optimized capillary fixation (critical to the acoustic system performance) and upgrading of the circuit board which is likely to result in improved EV isolation results in forthcoming studies when using the latest version of the instrument.

EVs are constitutively released by all cell types under physiological and pathological conditions [19]. Here, we show that numbers of EVs increased in the majority of patients after allo-HSCT, but that EV numbers were not correlated with clinical complications. This is likely due to the fact that a number of changes in important clinical parameters and medications occur concurrently, and that a change of EV numbers as a single parameter reflects the sum of the different perturbations rather than a specific complication.

On the other hand, allo-HSCT affected EV cargo as indicated by miRNA expression pattern changes across different time points. This motivated us to further investigate if plasma EV miRNA profiles correlated to transplantation-related complication. We identified miRNAs that positively correlated to infection and, interestingly, all of the top five have been previously reported to play a role in regulating inflammatory processes in different disease settings [30–34]. miR-21 expression was shown to be induced by inflammatory stimuli [35] and its expression is correlated with C-reactive protein and fibrinogen levels [36], which indicates that EV miR-21 is worth to be further investigated as potential biomarker for infection/inflammation. With regard to the identified downregulated miRNAs, only miR-320b downregulation has been reported in the context of infection [37], suggesting that miR-320b may regulate immune pathway target genes in allo-HSCT. To our knowledge, none of the other identified EV miRNAs have been reported in the context of infection pathogenesis and further investigation of their possible role in infection pathophysiology is therefore motivated.

GvHD is an immune-mediated disease and dysregulation of miRNAs in immune cells is associated with GvHD pathophysiology [38]. Accordingly, the top five positively correlated GvHD miRNAs identified herein have been demonstrated to have critical immune-regulatory roles in different contexts. For instance, miR-15b-3p as regulator of CD4+ regulatory T cell development [39], miR-342-3p in acute renal transplant rejection, and miR-130a-3p and miR-145-5p as controlling macrophage properties [40–42]. More importantly, expression of miR-30a-5p was upregulated in aGvHD patients but did not correlate with aGvHD severity [4]. In addition, we also identified GvHD-associated miR-155-5p which is upregulated in the plasma and in the gut of aGVHD patients [4, 43]. Thus, miR-155-5p in EVs may represent a more specific GvHD biomarker and potential target for GvHD treatment, which is certainly worth to be investigated further.

It has been demonstrated that expression of miR-93*/miR-93-3p together with miR-423, miR-199a-3p and miR-377 in the plasma was able to predict the probability of aGvHD occurrence and was furthermore positively associated with disease severity and patient survival [4]. Importantly, high expression of these miRNAs was detectable before GvHD was diagnosed clinically [4, 44]. However, our data did not show a positive correlation of miR-93* with GvHD status but instead we found that miR-93-5p, which is another strand of miR-93, was negatively correlated with GvHD. Also, we observed a negative correlation of miR-18a-5p expression in EVs in contrast with published data on increased miR-18a-5p levels in the serum of aGvHD patients [45]. This discrepancy between miRNA expression in whole plasma versus EVs remains unclear but might be explained with the reported selective sorting of miRNAs into the EVs [46] as also suggested by our data (Fig. 3).

Interestingly, from the correlation analysis data it was intriguing to observe that miR-342-3p was regulated differently in GvHD and infection, indicating that this miRNAs could play different roles in the different disease states. However, these data have to be further confirmed and validated in a larger independent cohort.

Due to the design of the study, which was limited to a 3-month observation time post transplantation and of which all of the patients survived, we could not evaluate a possible correlation between miRNA expression and survival. Interestingly, however, we found that peripheral blood EV miR-128 expression preceded the detection of relapse in a patient with ALL by several weeks. As miR-128 has been reported to be associated with ALL [47] and ALL cells have been reported to produce EVs [48], we hypothesize that ALL-specific EVs might be used for non-invasive remission monitoring, and, accordingly, we have initiated a biomarker study in ALL patients addressing this important question.

## Conclusions

In this study, we have demonstrated that acoustic trapping efficiently and rapidly enriched EVs from minute sample volumes of patient plasma. EVs enriched by acoustic trapping contained amounts and qualities of RNA that were sufficient to perform downstream miRNA analysis. More importantly, acoustically enriched EVs contained miRNAs that correlated with clinical complications, specifically infection and GvHD, and thus could potentially be further developed as non-invasive diagnostic biomarkers in allo-HSCT. Based on these proof-of-priciple data, we therefore plan to conduct larger biomarker discovery and validation studies aiming to identify reliable predictive EV biomarker in transplantation, e.g. by applying machine learning (Artificial Intelligence) strategies. In summary, we propose that acoustic EV trapping has the potential to become a novel clinical tool to rapidly enrich EVs in minute plasma volumes from larger patient cohorts for diagnostic and prognostic purposes not only in hematopoietic stem cell transplantation but also in other transplantation settings (e.g. organ transplant rejection) as well as a broad range of other diseases.

## Supplementary Information

**Additional file 1.**

## Data Availability

The datasets supporting the conclusions of this article are included within the article and supplementary files.

## Abbreviations

Allo-HSCT: Allogeneic hematopoietic stem cell transplantation
CRP: C-reactive protein
EV: Extracellular vesicle
GvHD: Graft-versus-host disease
miRNA: MicroRNA
qPCR: Quantitative polymerase chain reaction
TEM: Transmission electron microscopy
