# Supplementary material for: Development of acoustically isolated extracellular plasma vesicles for biomarker discovery in allogeneic hematopoietic stem cell transplantation
Source: Biomark Res. 2021 Jan 19;9:6. doi: 10.1186/s40364-020-00259-4 (PMC7814576; doi:10.1186/s40364-020-00259-4)
Supplement: Supplementary file 1 — Additional file 1. [file 40364_2020_259_MOESM1_ESM.pdf]

Supplementary Table 1: All coefficient correlation values for miRNA correlated with infectious complication

miR name	Pat 1	Pat 2	Pat 5	Pat 6	Pat 7	Pat 8	Pat 9	Pat 11	Pat 12	Pat 13	Pat 14	Pat 16	Pat 19	Pat 20
hsa-miR-223-3p	0.000000	0.621059	0.414039	0.654654	0.654654	0.654654	0.828079	0.000000	0.487950	0.392792	0.392792	0.878310	0.828079	0.654654
hsa-miR-21-5p	0.392792	0.828079	-0.828079	0.654654	0.392792	0.654654	0.414039	-0.414039	0.292770	0.130931	0.654654	0.396059	0.621059	0.654654
hsa-miR-23a-3p	-0.392792	0.828079	-0.621059	0.654654	-0.392792	0.654654	0.828079	0.414039	-0.292770	0.654654	0.392792	0.683130	0.621059	0.654654
hsa-miR-375	1.000000	0.122474	0.000000	0.000000	-0.200000	0.000000	0.632456	0.979796	0.000000	-0.200000	0.000000	0.447214	-0.489898	1.000000
hsa-miR-423-5p	-0.130931	0.828079	0.207020	-0.531369	0.654654	0.392792	0.840168	-0.316228	0.447214	0.139122	0.464758	-0.447214	-0.219971	0.139122
hsa-miR-25-3p	0.130931	0.828079	-0.621059	0.392792	0.392792	0.654654	0.621059	0.621059	-0.292770	-0.392792	-0.654654	0.495074	-0.414039	0.392792
hsa-miR-106a-5p	0.392792	0.828079	-0.414039	0.392792	0.654654	-0.130931	0.828079	0.000000	-0.891133	0.392792	0.392792	-0.495074	-0.621059	0.654654
hsa-miR-486-5p	0.774597	0.219971	-0.367423	0.464758	0.695608	0.464758	0.000000	0.612372	-0.577350	-0.417365	0.200000	0.396059	-0.840168	0.132842
hsa-let-7c-5p	-0.130931	0.414039	-0.207020	0.654654	0.654654	0.392792	-0.207020	0.659912	-0.292770	-0.130931	0.130931	-0.311086	-0.621059	0.654654
hsa-miR-2110	0.139122	-0.659912	0.316228	-0.200000	-0.309839	0.774597	0.367423	-0.316228	0.692820	-0.200000	-0.200000	0.000000	0.000000	1.000000
hsa-miR-151a-5p	-0.531369	0.828079	0.414039	0.392792	0.654654	-0.200000	-0.315063	0.000000	-0.396059	0.392792	-0.531369	0.000000	0.000000	0.654654
hsa-miR-24-3p	0.130931	-0.414039	0.000000	0.654654	0.130931	0.654654	0.414039	0.659912	-0.396059	-0.130931	-0.392792	0.000000	-0.414039	0.392792
hsa-miR-20a-5p	0.392792	0.621059	-0.414039	0.654654	0.392792	0.392792	0.414039	0.219971	-0.683130	-0.654654	0.130931	-0.292770	-0.621059	0.654654
hsa-miR-152-3p	0.000000	0.000000	-0.659912	0.464758	0.000000	1.000000	0.659912	0.000000	0.115470	-0.200000	-0.200000	0.692820	-0.316228	-0.417365
hsa-miR-505-3p	0.000000	0.000000	-0.367423	-0.200000	0.139122	0.464758	0.000000	0.000000	0.447214	0.139122	-0.200000	0.518476	0.000000	0.139122
hsa-miR-122-5p	-0.392792	0.828079	0.000000	-0.130931	0.695608	-0.130931	-0.105021	0.632456	0.103695	0.139122	-0.417365	0.692820	-0.659912	-0.200000
hsa-let-7e-5p	0.130931	0.000000	0.207020	-0.130931	0.130931	0.695608	-0.219971	0.000000	0.000000	-0.130931	-0.417365	0.115470	0.000000	0.654654
hsa-miR-502-3p	0.000000	-0.219971	0.000000	1.000000	-0.200000	0.000000	0.000000	0.000000	0.000000	-0.200000	0.000000	0.000000	0.632456	0.000000
hsa-miR-15b-5p	-0.654654	0.828079	0.207020	0.392792	0.654654	-0.130931	0.828079	-0.489898	0.103695	-0.130931	-0.654654	-0.103695	0.000000	0.130931
hsa-miR-195-5p	0.000000	0.979796	0.000000	0.000000	0.000000	0.000000	0.000000	0.000000	-0.692820	0.417365	-0.309839	-0.447214	0.632456	0.392792
hsa-miR-144-3p	0.654654	0.414039	-0.414039	-0.130931	0.392792	-0.130931	0.659912	0.000000	0.447214	0.417365	-0.531369	-0.115470	-0.219971	-0.654654
hsa-miR-15a-5p	-0.130931	0.621059	-0.414039	0.654654	0.654654	-0.130931	0.828079	0.000000	-0.115470	-0.417365	-0.654654	-0.103695	-0.414039	0.392792
hsa-miR-133b	0.139122	-0.316228	-0.367423	0.000000	0.000000	0.000000	-0.489898	0.000000	0.000000	1.000000	0.000000	0.447214	0.632456	-0.309839
hsa-miR-23b-3p	-0.654654	0.828079	0.000000	0.654654	-0.654654	-0.130931	0.414039	0.207020	-0.878310	0.654654	0.654654	-0.487950	-0.621059	0.654654
hsa-miR-103a-3p	-0.531369	0.414039	0.000000	-0.130931	0.392792	0.654654	0.621059	-0.316228	-0.518476	0.392792	0.654654	0.198030	-0.621059	-0.654654
hsa-miR-660-5p	0.000000	0.632456	0.489898	-0.417365	0.000000	-0.417365	0.122474	0.000000	0.000000	1.000000	-0.200000	0.000000	-0.489898	-0.200000
hsa-miR-92a-3p	0.130931	0.828079	-0.207020	0.654654	0.654654	-0.392792	0.000000	0.414039	-0.683130	-0.654654	-0.130931	-0.097590	-0.414039	0.392792
hsa-miR-324-3p	-0.309839	-0.316228	-0.367423	-0.309839	0.417365	0.417365	0.659912	0.000000	-0.115470	0.000000	0.000000	0.447214	0.122474	-0.200000

hsa-miR-543	0.000000	0.000000	0.000000	-0.200000	-0.200000	0.000000	0.000000	0.000000	-0.447214	0.000000	1.000000	0.447214	0.000000	-0.200000
hsa-miR-20b-5p	0.000000	0.000000	-0.367423	-0.200000	0.000000	0.000000	0.632456	0.000000	0.447214	0.000000	-0.200000	0.000000	0.367423	-0.309839
hsa-miR-362-3p	0.000000	0.000000	0.316228	0.000000	0.000000	0.000000	0.000000	0.000000	0.000000	0.000000	0.000000	0.000000	0.000000	0.000000
hsa-miR-26a-5p	-0.654654	0.000000	0.828079	0.130931	0.130931	0.392792	0.414039	-0.828079	-0.292770	0.392792	-0.130931	-0.487950	0.000000	0.392792
hsa-let-7f-5p	-0.654654	0.828079	-0.828079	0.654654	-0.531369	0.654654	0.621059	0.000000	0.000000	-0.417365	0.000000	0.000000	0.439941	-0.531369
hsa-miR-100-5p	-0.200000	0.122474	0.000000	-0.200000	0.000000	0.000000	-0.316228	-0.489898	0.447214	0.000000	0.464758	0.692820	-0.316228	0.000000
hsa-miR-29b-3p	0.000000	0.000000	0.000000	0.000000	0.000000	0.000000	0.000000	0.000000	0.000000	0.000000	0.000000	0.000000	0.000000	0.200000
hsa-miR-19b-3p	-0.654654	-0.207020	-0.828079	0.654654	0.392792	0.392792	-0.207020	0.367423	-0.396059	-0.130931	0.654654	-0.292770	-0.207020	0.654654
hsa-miR-629-5p	-0.200000	0.000000	0.489898	0.000000	-0.309839	0.000000	0.632456	0.000000	0.000000	0.000000	0.000000	-0.447214	0.000000	0.000000
hsa-miR-93-3p	-0.200000	0.000000	-0.632456	0.000000	0.000000	-0.200000	0.000000	0.000000	0.000000	0.774597	0.000000	0.000000	0.420084	0.000000
hsa-miR-7-1-3p	0.000000	0.000000	-0.632456	0.000000	0.000000	0.000000	0.000000	0.659912	0.000000	0.417365	0.000000	0.000000	0.000000	-0.309839
hsa-miR-126-5p	-0.392792	0.828079	0.000000	-0.130931	0.392792	-0.531369	0.207020	0.207020	-0.878310	-0.654654	0.392792	0.292770	0.000000	0.392792
hsa-let-7a-5p	-0.654654	0.828079	-0.828079	0.392792	0.392792	0.654654	0.414039	-0.489898	-0.683130	0.392792	0.130931	-0.292770	-0.828079	0.654654
hsa-miR-365a-3p	-0.200000	-0.316228	-0.122474	0.000000	0.000000	1.000000	-0.316228	0.000000	0.000000	0.000000	0.000000	0.000000	0.000000	0.000000
hsa-miR-142-3p	-0.392792	0.828079	-0.621059	0.654654	0.654654	0.130931	0.207020	-0.219971	-0.198030	-0.654654	-0.531369	-0.692820	0.207020	0.654654
hsa-miR-126-3p	0.000000	0.000000	0.000000	0.000000	0.000000	0.000000	0.000000	0.000000	0.000000	0.000000	0.000000	0.000000	0.000000	0.000000
hsa-miR-107	0.654654	0.828079	-0.828079	0.654654	-0.654654	0.654654	0.105021	-0.316228	-0.518476	-0.417365	-0.417365	-0.447214	0.000000	0.654654
hsa-miR-590-5p	0.000000	0.000000	-0.367423	-0.200000	1.000000	0.000000	0.000000	0.632456	0.000000	0.132842	-0.200000	-0.447214	-0.489898	-0.200000
hsa-miR-130b-3p	-0.200000	-0.525105	0.000000	-0.309839	0.000000	0.000000	0.632456	0.000000	0.000000	-0.200000	0.000000	0.000000	0.632456	-0.200000
hsa-miR-874-3p	-0.200000	0.439941	0.489898	-0.417365	-0.200000	-0.309839	0.219971	0.000000	0.447214	0.000000	-0.200000	-0.447214	0.000000	0.000000
hsa-miR-136-3p	0.000000	0.000000	0.000000	0.000000	-0.200000	0.000000	0.000000	0.000000	0.000000	0.000000	0.000000	0.000000	0.000000	0.000000
hsa-miR-495-3p	0.000000	0.000000	0.000000	-0.309839	0.000000	0.000000	0.000000	0.000000	0.000000	0.000000	0.000000	0.000000	0.000000	0.000000
hsa-miR-485-3p	0.000000	0.632456	-0.632456	-0.309839	0.000000	0.000000	0.000000	0.000000	0.000000	0.000000	0.000000	0.000000	0.000000	0.000000
hsa-miR-200a-3p	0.000000	0.000000	0.000000	0.000000	0.000000	0.000000	0.000000	0.000000	0.000000	0.000000	0.000000	0.000000	-0.316228	0.000000
hsa-miR-133a-3p	-0.392792	-0.105021	0.000000	0.464758	-0.200000	-0.309839	0.659912	0.000000	-0.447214	0.000000	0.000000	0.000000	0.000000	0.000000
hsa-miR-154-5p	0.000000	0.000000	0.000000	-0.200000	0.000000	0.000000	0.000000	0.000000	0.000000	0.000000	0.000000	0.000000	0.000000	-0.200000
hsa-miR-320a	-0.130931	-0.207020	0.000000	0.654654	0.130931	0.392792	0.414039	0.000000	-0.878310	-0.654654	-0.130931	0.198030	-0.621059	0.392792
hsa-miR-421	0.000000	0.000000	-0.979796	0.000000	0.000000	0.000000	0.000000	0.632456	0.000000	-0.531369	0.000000	0.000000	0.632456	-0.200000
hsa-miR-324-5p	0.000000	0.000000	-0.122474	-0.309839	-0.200000	0.000000	0.632456	0.000000	0.000000	0.000000	0.000000	-0.447214	0.000000	0.000000
hsa-miR-125b-5p	-0.654654	0.000000	0.489898	-0.132842	-0.417365	-0.392792	0.420084	-0.659912	-0.518476	-0.417365	0.774597	0.725866	0.420084	-0.132842

hsa-let-7i-5p	-0.654654	0.414039	-0.414039	0.392792	0.130931	0.654654	0.630126	0.367423	-0.891133	-0.132842	-0.309839	0.115470	-0.414039	-0.392792
hsa-miR-574-3p	-0.309839	-0.316228	-0.632456	-0.200000	0.000000	0.000000	0.367423	0.979796	0.447214	-0.417365	0.000000	0.000000	-0.316228	-0.200000
hsa-miR-223-5p	0.000000	0.000000	0.000000	-0.200000	0.000000	0.000000	0.000000	0.000000	0.000000	-0.200000	0.000000	0.000000	0.000000	-0.200000
hsa-miR-497-5p	-0.309839	0.000000	-0.632456	-0.200000	1.000000	0.000000	-0.316228	0.000000	0.000000	0.000000	0.000000	0.000000	0.000000	-0.200000
hsa-miR-885-5p	0.000000	0.000000	0.000000	0.000000	0.000000	0.000000	-0.316228	0.000000	0.000000	-0.200000	-0.200000	0.000000	0.000000	0.000000
hsa-miR-155-5p	-0.200000	0.367423	0.489898	-0.309839	0.000000	0.000000	0.000000	0.000000	0.000000	-0.309839	-0.417365	0.000000	0.000000	-0.417365
hsa-miR-199a-3p	0.139122	-0.414039	0.414039	-0.417365	0.392792	-0.309839	0.219971	0.000000	0.000000	-0.417365	0.000000	0.000000	0.000000	-0.417365
hsa-miR-27a-3p	-0.654654	0.000000	-0.621059	0.392792	-0.531369	-0.654654	-0.207020	0.122474	-0.396059	0.654654	0.392792	-0.198030	0.207020	0.654654
hsa-miR-106b-5p	-0.654654	0.000000	-0.621059	0.654654	0.392792	-0.654654	-0.105021	0.367423	-0.115470	0.139122	-0.654654	-0.292770	0.000000	0.654654
hsa-miR-501-3p	0.000000	0.000000	0.000000	-0.200000	-0.200000	0.000000	0.000000	0.000000	0.000000	-0.200000	0.000000	0.000000	0.000000	-0.309839
hsa-miR-28-3p	-0.265684	-0.659912	-0.105021	-0.132842	0.130931	-0.132842	0.621059	0.439941	-0.198030	-0.531369	-0.132842	0.099015	-0.489898	0.398527
hsa-miR-128-3p	-0.200000	-0.316228	-0.367423	0.000000	0.000000	0.000000	0.122474	0.000000	0.000000	0.000000	-0.200000	0.000000	0.000000	0.000000
hsa-miR-16-5p	-0.392792	0.414039	-0.621059	0.654654	0.654654	-0.392792	0.828079	0.000000	-0.878310	-0.654654	-0.130931	0.097590	-0.414039	-0.130931
hsa-miR-200c-3p	0.139122	-0.316228	0.000000	-0.309839	-0.309839	0.000000	0.122474	0.000000	0.000000	0.139122	0.000000	0.000000	-0.316228	-0.132842
hsa-miR-326	0.000000	-0.316228	0.316228	-0.417365	0.139122	-0.200000	-0.316228	0.000000	0.000000	0.000000	0.000000	0.000000	0.000000	-0.200000
hsa-miR-30a-5p	-0.417365	-0.828079	-0.105021	-0.309839	-0.200000	-0.309839	-0.489898	0.000000	0.000000	0.392792	-0.309839	0.518476	0.840168	0.139122
hsa-miR-26b-5p	-0.130931	-0.207020	-0.207020	0.654654	0.654654	0.130931	0.000000	-0.489898	-0.396059	-0.392792	0.130931	-0.692820	-0.621059	0.392792
hsa-miR-532-3p	-0.200000	0.000000	0.000000	0.000000	-0.200000	0.000000	-0.316228	0.000000	0.000000	-0.417365	0.000000	0.000000	0.122474	-0.200000
hsa-miR-32-5p	-0.309839	0.632456	-0.367423	-0.417365	0.000000	-0.200000	-0.489898	-0.316228	-0.447214	0.000000	0.000000	0.000000	0.632456	0.000000
hsa-miR-16-2-3p	-0.200000	0.632456	-0.414039	-0.309839	-0.200000	0.000000	-0.316228	-0.316228	0.000000	0.774597	-0.531369	0.000000	-0.219971	-0.200000
hsa-let-7b-3p	-0.392792	0.828079	-0.420084	0.130931	-0.132842	-0.130931	-0.315063	0.122474	-0.396059	0.132842	-0.392792	-0.447214	0.439941	-0.392792
hsa-miR-532-5p	0.000000	0.000000	0.000000	-0.200000	0.000000	0.000000	0.000000	0.000000	0.000000	-0.417365	0.000000	0.000000	-0.489898	-0.309839
hsa-miR-425-3p	0.000000	-0.316228	0.000000	-0.200000	0.000000	0.000000	0.122474	0.000000	0.000000	-0.309839	-0.200000	0.000000	-0.316228	-0.200000
hsa-miR-141-3p	-0.309839	-0.316228	0.316228	-0.200000	0.000000	-0.309839	0.000000	-0.316228	0.000000	0.000000	0.000000	0.000000	-0.316228	0.000000
hsa-miR-27b-3p	0.392792	-0.621059	-0.420084	-0.654654	-0.392792	-0.531369	0.207020	0.219971	-0.683130	0.654654	-0.654654	0.292770	0.828079	-0.130931
hsa-miR-17-5p	-0.130931	0.621059	0.000000	0.130931	0.392792	0.130931	0.000000	-0.316228	-0.933257	0.392792	-0.654654	-0.097590	-0.414039	-0.654654
hsa-miR-125a-5p	-0.654654	-0.414039	0.000000	-0.531369	-0.132842	-0.654654	0.000000	-0.219971	0.000000	0.139122	0.654654	-0.292770	0.414039	0.139122
hsa-miR-34a-5p	0.000000	-0.316228	-0.105021	-0.309839	-0.200000	-0.309839	0.000000	0.000000	0.000000	0.000000	0.000000	0.000000	-0.316228	0.000000
hsa-miR-382-5p	0.000000	0.000000	-0.632456	-0.309839	0.000000	0.000000	0.000000	0.000000	-0.447214	-0.417365	0.000000	0.447214	0.000000	-0.200000
hsa-miR-140-5p	-0.200000	-0.489898	-0.219971	-0.309839	0.000000	-0.200000	0.000000	0.000000	0.000000	-0.200000	0.000000	0.000000	0.367423	-0.309839

hsa-miR-409-3p	0.000000	-0.316228	-0.219971	-0.309839	0.000000	0.000000	-0.316228	0.632456	-0.447214	-0.200000	-0.200000	0.000000	0.000000	-0.200000
hsa-miR-210-3p	-0.309839	-0.219971	-0.414039	-0.309839	-0.200000	0.000000	0.122474	0.000000	0.000000	0.000000	0.000000	0.000000	-0.316228	0.000000
hsa-miR-132-3p	0.000000	0.632456	0.000000	-0.309839	-0.309839	0.000000	-0.316228	0.000000	-0.115470	0.000000	0.000000	-0.447214	-0.659912	-0.200000
hsa-miR-143-3p	-0.309839	-0.489898	-0.828079	-0.417365	0.000000	-0.309839	0.367423	0.439941	0.097590	-0.130931	-0.139122	-0.097590	-0.315063	0.398527
hsa-miR-127-3p	-0.200000	-0.316228	0.000000	-0.200000	-0.200000	-0.200000	0.000000	0.000000	0.000000	-0.309839	0.000000	0.000000	-0.316228	0.000000
hsa-miR-339-5p	1.000000	0.000000	-0.979796	-0.417365	-0.309839	-0.417365	0.367423	0.000000	0.000000	-0.200000	-0.417365	0.000000	0.000000	-0.417365
hsa-miR-339-3p	-0.200000	0.000000	-0.979796	-0.200000	0.000000	0.000000	-0.316228	-0.316228	0.000000	0.000000	0.000000	-0.115470	0.632456	-0.309839
hsa-miR-1260a	-0.654654	0.000000	-0.207020	-0.392792	0.392792	0.130931	0.207020	-0.207020	-0.878310	-0.392792	-0.130931	0.292770	0.414039	-0.392792
hsa-let-7g-5p	-0.654654	0.000000	-0.828079	0.654654	0.654654	0.130931	0.000000	0.367423	-0.447214	-0.531369	-0.130931	-0.447214	-0.207020	-0.392792
hsa-miR-424-5p	-0.309839	-0.489898	-0.840168	-0.309839	-0.200000	0.139122	0.367423	0.000000	0.000000	0.000000	0.000000	0.000000	0.000000	-0.200000
hsa-miR-451a	-0.130931	0.207020	-0.621059	-0.130931	0.654654	-0.654654	0.414039	-0.207020	-0.878310	-0.654654	0.392792	0.292770	-0.414039	-0.130931
hsa-miR-29c-3p	-0.531369	-0.414039	0.000000	0.130931	-0.309839	0.417365	0.000000	0.000000	0.000000	0.000000	-0.309839	-0.115470	-0.315063	-0.417365
hsa-miR-652-3p	-0.417365	0.219971	0.000000	-0.654654	-0.417365	-0.392792	0.000000	0.000000	0.000000	-0.417365	0.000000	0.447214	0.122474	-0.417365
hsa-miR-186-5p	-0.200000	0.000000	-0.979796	-0.200000	0.000000	-0.200000	0.000000	0.000000	-0.447214	-0.200000	-0.200000	0.000000	0.000000	0.464758
hsa-miR-29a-3p	-0.200000	0.000000	-0.367423	-0.531369	0.392792	-0.309839	0.632456	0.000000	0.000000	-0.417365	-0.200000	0.000000	-0.659912	-0.309839
hsa-miR-19a-3p	0.130931	0.207020	-0.414039	-0.654654	0.654654	0.130931	0.000000	-0.489898	-0.487950	0.392792	-0.130931	-0.103695	-0.828079	-0.392792
hsa-miR-363-3p	-0.200000	-0.489898	-0.367423	-0.309839	-0.531369	0.000000	0.000000	0.000000	0.000000	0.000000	-0.309839	0.000000	0.420084	-0.200000
hsa-miR-199a-5p	-0.200000	-0.316228	0.000000	-0.417365	-0.309839	-0.200000	0.367423	-0.316228	0.000000	-0.200000	-0.200000	0.115470	-0.316228	0.000000
hsa-miR-194-5p	0.000000	-0.489898	0.659912	-0.531369	-0.200000	-0.132842	0.000000	0.632456	-0.447214	-0.417365	0.132842	0.000000	-0.828079	-0.417365
hsa-miR-92b-3p	0.000000	0.000000	0.000000	-0.200000	0.000000	-0.200000	0.000000	0.000000	-0.447214	-0.200000	-0.200000	-0.115470	-0.489898	-0.200000
hsa-miR-33a-5p	-0.309839	0.000000	-0.420084	-0.417365	0.000000	-0.309839	0.219971	0.000000	0.000000	0.000000	-0.200000	0.000000	-0.316228	-0.309839
hsa-miR-145-5p	-0.392792	0.000000	-0.414039	-0.531369	0.654654	-0.531369	0.219971	0.122474	0.000000	-0.392792	-0.531369	0.447214	-0.207020	-0.531369
hsa-miR-320d	-0.417365	0.210042	0.105021	-0.417365	-0.132842	-0.309839	-0.316228	-0.316228	-0.878310	0.664211	-0.417365	0.518476	0.000000	-0.392792
hsa-miR-22-5p	0.000000	-0.659912	-0.632456	-0.417365	0.000000	-0.200000	-0.316228	0.000000	0.115470	0.000000	0.000000	0.000000	0.000000	0.000000
hsa-miR-338-3p	0.139122	-0.840168	-0.207020	0.139122	-0.417365	-0.309839	0.219971	0.000000	0.000000	-0.200000	0.000000	0.000000	-0.659912	0.000000
hsa-miR-22-3p	-0.417365	0.105021	0.000000	-0.130931	-0.130931	-0.654654	0.000000	-0.316228	0.000000	-0.200000	-0.200000	0.000000	0.210042	-0.417365
hsa-miR-376c-3p	-0.200000	-0.489898	-0.219971	-0.654654	-0.309839	-0.200000	-0.316228	0.000000	0.000000	-0.200000	1.000000	0.000000	-0.316228	-0.309839
hsa-miR-28-5p	0.000000	-0.525105	0.105021	-0.531369	-0.200000	0.000000	-0.316228	0.000000	0.000000	-0.309839	0.000000	-0.447214	0.420084	-0.417365
hsa-miR-423-3p	-0.200000	-0.489898	0.207020	-0.531369	-0.309839	-0.200000	-0.659912	0.000000	0.447214	0.000000	0.000000	-0.447214	0.367423	-0.417365
hsa-miR-192-5p	-0.417365	-0.316228	0.000000	-0.309839	0.464758	-0.417365	-0.489898	0.122474	-0.447214	-0.132842	-0.417365	0.000000	0.122474	0.000000

hsa-miR-136-5p	-0.200000	0.000000	0.489898	-0.417365	-0.200000	-0.200000	-0.316228	-0.489898	0.000000	-0.200000	-0.200000	0.000000	-0.316228	-0.200000
hsa-miR-877-5p	0.000000	-0.316228	0.000000	-0.200000	0.000000	-0.200000	0.000000	0.000000	-0.115470	-0.200000	-0.309839	-0.447214	0.000000	-0.531369
hsa-miR-483-5p	0.139122	-0.316228	-0.367423	-0.200000	-0.309839	-0.309839	-0.316228	-0.207020	0.292770	-0.130931	0.130931	0.487950	-0.828079	-0.392792
hsa-miR-215-5p	-0.200000	-0.489898	-0.879883	-0.200000	-0.309839	-0.417365	0.000000	0.000000	0.447214	-0.309839	0.000000	0.447214	-0.219971	-0.200000
hsa-miR-191-5p	-0.654654	-0.414039	-0.207020	-0.654654	0.695608	-0.531369	0.828079	-0.659912	-0.297044	-0.130931	-0.654654	0.292770	-0.621059	0.654654
hsa-miR-376a-3p	-0.200000	-0.414039	-0.367423	-0.531369	-0.309839	0.139122	0.210042	0.000000	0.000000	-0.200000	0.000000	0.000000	-0.316228	-0.417365
hsa-miR-181a-5p	0.417365	0.105021	-0.659912	-0.654654	-0.417365	-0.309839	-0.525105	0.000000	0.447214	-0.309839	-0.309839	-0.447214	0.122474	0.132842
hsa-miR-1	0.000000	-0.659912	-0.122474	-0.309839	0.000000	-0.200000	0.000000	-0.525105	-0.097590	-0.417365	-0.309839	0.518476	0.219971	-0.531369
hsa-miR-106b-3p	-0.200000	-0.489898	-0.632456	-0.200000	0.132842	-0.200000	-0.840168	0.000000	0.000000	-0.200000	0.000000	-0.447214	0.840168	-0.200000
hsa-miR-484	-0.654654	0.207020	-0.621059	-0.531369	-0.531369	-0.130931	0.659912	0.122474	0.103695	-0.417365	-0.130931	0.099015	-0.621059	-0.132842
hsa-miR-30e-5p	-0.654654	-0.207020	0.000000	-0.654654	-0.417365	0.417365	0.630126	-0.316228	0.000000	-0.309839	-0.200000	-0.447214	0.207020	-0.654654
hsa-miR-193a-5p	-0.132842	-0.840168	-0.367423	-0.392792	-0.309839	-0.417365	0.367423	0.632456	-0.447214	0.464758	-0.200000	-0.933257	0.367423	-0.417365
hsa-miR-30c-5p	-0.531369	0.828079	-0.621059	0.392792	-0.531369	-0.417365	0.000000	-0.659912	-0.495074	0.398527	-0.130931	0.000000	-0.828079	-0.130931
hsa-miR-584-5p	-0.309839	0.000000	-0.630126	-0.417365	-0.200000	-0.200000	0.367423	0.122474	-0.198030	0.417365	-0.695608	-0.577350	0.000000	-0.417365
hsa-miR-197-3p	-0.200000	0.632456	-0.630126	-0.654654	0.000000	-0.417365	0.439941	-0.316228	-0.692820	0.130931	-0.309839	0.000000	-0.315063	-0.417365
hsa-miR-101-3p	-0.654654	-0.105021	-0.828079	0.398527	-0.417365	-0.654654	0.000000	0.000000	0.115470	0.417365	-0.531369	0.447214	-0.828079	-0.130931
hsa-miR-185-5p	-0.130931	-0.621059	-0.621059	-0.654654	0.654654	-0.392792	0.000000	0.000000	-0.683130	-0.392792	-0.654654	0.198030	-0.207020	0.654654
hsa-miR-335-5p	-0.309839	-0.840168	0.000000	-0.417365	0.000000	-0.200000	0.367423	0.000000	-0.447214	-0.309839	-0.200000	0.000000	-0.316228	-0.200000
hsa-miR-10b-5p	-0.531369	-0.828079	-0.219971	-0.417365	0.774597	-0.417365	0.219971	0.000000	-0.447214	-0.417365	-0.200000	-0.447214	0.367423	-0.309839
hsa-miR-144-5p	-0.417365	0.105021	-0.414039	-0.654654	-0.531369	0.417365	0.219971	-0.489898	-0.115470	-0.309839	-0.130931	-0.447214	0.420084	-0.531369
hsa-miR-148b-3p	-0.309839	-0.659912	-0.207020	-0.654654	-0.309839	-0.200000	0.367423	0.632456	-0.447214	-0.309839	0.000000	-0.447214	-0.219971	-0.200000
hsa-miR-301a-3p	-0.200000	0.000000	-0.105021	-0.417365	0.000000	-0.200000	-0.316228	0.000000	-0.447214	-0.417365	-0.309839	0.000000	-0.219971	-0.417365
hsa-miR-222-3p	-0.531369	-0.659912	-0.828079	-0.417365	-0.309839	0.392792	0.414039	0.000000	0.000000	-0.200000	0.000000	-0.447214	-0.219971	-0.309839
hsa-miR-766-3p	0.000000	0.000000	-0.367423	-0.200000	-0.200000	0.000000	0.000000	-0.316228	-0.577350	-0.309839	-0.417365	-0.115470	-0.316228	-0.309839
hsa-miR-221-3p	-0.417365	-0.659912	0.621059	-0.654654	-0.654654	0.392792	0.367423	-0.316228	-0.447214	-0.200000	-0.309839	-0.447214	-0.316228	-0.132842
hsa-miR-140-3p	-0.654654	0.840168	-0.621059	-0.392792	-0.417365	-0.654654	0.414039	-0.219971	-0.518476	0.130931	-0.531369	0.292770	-0.207020	-0.654654
hsa-miR-15b-3p	-0.309839	-0.316228	-0.367423	-0.309839	0.398527	-0.309839	0.000000	0.000000	-0.577350	-0.200000	0.000000	-0.447214	-0.489898	-0.309839
hsa-miR-331-3p	-0.309839	0.000000	-0.630126	-0.531369	0.000000	-0.200000	-0.489898	0.000000	0.000000	0.000000	0.000000	-0.447214	-0.489898	-0.200000
hsa-miR-30b-5p	-0.654654	-0.828079	-0.207020	-0.654654	-0.130931	-0.654654	0.414039	-0.414039	-0.487950	0.654654	-0.392792	-0.198030	-0.414039	0.654654
hsa-miR-320c	0.392792	0.000000	0.000000	-0.654654	-0.654654	-0.654654	0.207020	-0.207020	-0.878310	-0.654654	-0.654654	0.198030	-0.414039	0.654654

hsa-miR-18a-5p	-0.417365	-0.414039	-0.414039	-0.531369	-0.417365	-0.309839	0.659912	0.000000	-0.447214	0.417365	-0.417365	0.000000	-0.525105	-0.531369
hsa-miR-150-5p	-0.654654	0.000000	-0.439941	-0.654654	-0.309839	-0.531369	-0.105021	0.210042	-0.297044	0.130931	-0.417365	0.000000	0.105021	-0.392792
hsa-miR-205-5p	-0.417365	0.439941	-0.840168	-0.531369	-0.392792	-0.130931	-0.207020	-0.316228	0.000000	0.000000	0.000000	-0.447214	-0.316228	-0.200000
hsa-miR-374b-5p	-0.309839	-0.828079	-0.207020	-0.654654	0.417365	-0.200000	0.000000	-0.316228	-0.396059	0.130931	-0.417365	-0.115470	0.000000	-0.531369
hsa-miR-18b-5p	-0.200000	-0.840168	-0.414039	-0.531369	-0.417365	-0.417365	0.630126	0.000000	-0.447214	-0.132842	-0.309839	0.000000	0.000000	-0.531369
hsa-miR-7-5p	-0.654654	-0.828079	-0.414039	-0.392792	0.392792	-0.654654	0.000000	0.000000	-0.447214	0.139122	-0.392792	-0.297044	0.414039	-0.531369
hsa-miR-139-5p	-0.531369	-0.828079	-0.207020	-0.531369	0.417365	-0.531369	-0.489898	-0.316228	-0.115470	0.130931	-0.417365	-0.115470	-0.315063	0.139122
hsa-miR-130a-3p	-0.417365	0.367423	0.207020	-0.531369	-0.392792	-0.309839	0.219971	-0.525105	-0.198030	-0.130931	-0.392792	-0.692820	-0.525105	-0.417365
hsa-miR-328-3p	-0.654654	0.105021	-0.420084	-0.309839	0.000000	-0.309839	-0.316228	-0.316228	-0.692820	-0.309839	-0.200000	0.000000	-0.315063	-0.130931
hsa-miR-30e-3p	-0.200000	-0.489898	-0.879883	-0.309839	0.000000	-0.309839	-0.316228	-0.316228	0.115470	-0.417365	-0.200000	-0.447214	0.420084	-0.531369
hsa-miR-146a-5p	-0.531369	-0.840168	-0.621059	-0.654654	0.392792	-0.392792	-0.105021	0.000000	-0.518476	0.695608	-0.417365	-0.103695	-0.414039	-0.531369
hsa-let-7b-5p	-0.654654	-0.207020	-0.207020	-0.654654	0.654654	-0.654654	0.207020	-0.105021	-0.878310	-0.654654	0.130931	-0.292770	-0.414039	-0.392792
hsa-miR-335-3p	-0.654654	-0.735147	0.000000	-0.531369	-0.531369	-0.309839	0.207020	-0.414039	-0.292770	-0.392792	-0.654654	0.097590	0.414039	-0.392792
hsa-miR-342-3p	-0.417365	-0.489898	-0.210042	-0.309839	-0.417365	-0.200000	-0.105021	-0.489898	-0.487950	-0.309839	-0.309839	-0.447214	0.207020	-0.392792
hsa-miR-146b-5p	-0.200000	-0.659912	-0.659912	-0.417365	-0.531369	-0.200000	0.122474	-0.316228	-0.447214	0.417365	-0.309839	-0.447214	-0.315063	-0.417365
hsa-let-7d-3p	-0.200000	-0.414039	-0.219971	-0.132842	-0.309839	-0.417365	0.659912	-0.489898	-0.933257	-0.531369	-0.531369	-0.891133	0.105021	-0.132842
hsa-miR-148a-3p	-0.531369	-0.659912	0.315063	-0.531369	-0.417365	-0.654654	0.207020	0.000000	-0.447214	0.132842	0.000000	-0.692820	-0.659912	-0.531369
hsa-miR-361-5p	-0.417365	-0.840168	-0.621059	-0.654654	-0.417365	-0.417365	0.122474	-0.219971	0.103695	-0.130931	-0.200000	-0.577350	0.414039	-0.654654
hsa-miR-151a-3p	-0.309839	-0.525105	-0.420084	-0.531369	-0.309839	0.000000	-0.207020	-0.659912	-0.518476	0.132842	-0.531369	-0.115470	0.000000	-0.531369
hsa-miR-454-3p	-0.417365	-0.489898	-0.630126	-0.200000	-0.200000	-0.200000	-0.489898	0.000000	0.000000	-0.132842	-0.417365	-0.692820	-0.315063	-0.531369
hsa-miR-374a-5p	0.000000	-0.525105	0.207020	-0.531369	-0.417365	-0.531369	0.219971	-0.489898	-0.692820	-0.531369	-0.417365	-0.115470	-0.414039	-0.654654
hsa-let-7d-5p	-0.417365	-0.414039	-0.207020	-0.654654	-0.654654	-0.417365	0.219971	-0.316228	-0.692820	0.132842	-0.130931	-0.311086	-0.414039	-0.654654
hsa-miR-30d-5p	-0.654654	-0.414039	-0.207020	-0.654654	-0.417365	-0.417365	0.000000	-0.219971	-0.518476	0.664211	-0.417365	-0.447214	-0.621059	-0.654654
hsa-miR-142-5p	-0.417365	-0.840168	-0.414039	-0.531369	-0.531369	-0.654654	0.219971	0.000000	-0.115470	-0.531369	-0.309839	-0.692820	0.000000	-0.531369
hsa-miR-320b	-0.392792	-0.207020	0.207020	-0.654654	-0.417365	-0.654654	-0.525105	-0.828079	-0.878310	-0.392792	-0.130931	-0.518476	-0.414039	-0.392792
hsa-miR-425-5p	-0.531369	-0.659912	-0.414039	-0.654654	-0.531369	-0.654654	-0.659912	0.000000	-0.692820	-0.531369	-0.200000	-0.311086	0.000000	-0.531369

Supplementary Table 2: All coefficient correlation values for miRNA correlated with GvHD

miR name	Pat 1	Pat 2	Pat 7	Pat 8
hsa-miR-145-5p	0.000000	0.547723	0.366679	0.722185
hsa-miR-15b-3p	0.250000	0.600000	0.429058	0.339656
hsa-miR-424-5p	0.108465	0.715097	0.800000	-0.009631
hsa-miR-30c-5p	0.278543	0.639010	-0.183340	0.712806
hsa-miR-130a-3p	0.200000	0.143019	0.730297	0.340926
hsa-miR-139-5p	0.733359	0.182574	0.429058	0.019263
hsa-miR-143-3p	0.488094	0.488094	0.500000	-0.169828
hsa-miR-361-5p	0.700000	0.371391	-0.371391	0.505076
hsa-miR-194-5p	0.000000	0.488094	0.000000	0.637774
hsa-miR-324-5p	0.500000	0.375000	0.250000	0.000000
hsa-miR-155-5p	0.500000	0.600000	0.000000	0.000000
hsa-miR-27a-3p	0.456435	0.547723	0.275010	-0.239756
hsa-miR-342-3p	0.464238	0.100000	0.333712	0.101015
hsa-miR-26a-5p	0.639010	-0.091287	0.091287	0.212092
hsa-miR-27b-3p	0.456435	0.000000	-0.456435	0.815976
hsa-miR-30a-5p	0.300000	0.182574	0.250000	0.040406
hsa-miR-409-3p	0.500000	0.250000	0.000000	0.000000
hsa-miR-221-3p	0.464238	0.278543	0.456435	-0.462308
hsa-let-7f-5p	0.456435	-0.365148	0.275010	0.350413
hsa-miR-320c	-0.273861	0.000000	0.456435	0.453743
hsa-miR-142-5p	0.000000	-0.229175	0.366679	0.481523
hsa-miR-100-5p	0.500000	0.108465	0.000000	0.000000
hsa-miR-502-3p	0.000000	0.100000	0.500000	0.000000
hsa-miR-99b-5p	0.108465	0.488094	0.000000	0.000000
hsa-miR-146a-5p	0.476731	-0.185695	-0.595854	0.866811
hsa-miR-374b-5p	0.300000	0.182574	-0.200000	0.227284
hsa-miR-154-5p	0.000000	0.000000	0.500000	0.000000
hsa-miR-197-3p	0.250000	0.375000	0.000000	-0.161624

hsa-miR-106b-5p	0.550019	-0.365148	0.273861	0.000000
hsa-let-7e-5p	0.182574	0.182574	0.365148	-0.282843
hsa-miR-15a-5p	-0.091287	-0.547723	0.547723	0.534841
hsa-miR-133a-3p	-0.286039	0.185695	0.500000	-0.012627
hsa-miR-1	0.000000	0.143019	0.000000	0.227284
hsa-miR-136-5p	0.500000	0.000000	0.125000	-0.303046
hsa-let-7b-5p	0.547723	-0.182574	-0.091287	0.046107
hsa-miR-140-3p	0.273861	0.275010	0.092848	-0.322749
hsa-miR-33a-5p	0.250000	0.375000	0.125000	-0.454569
hsa-miR-375	-0.250000	-0.750000	0.750000	0.530330
hsa-miR-484	0.273861	-0.273861	0.366679	-0.103169
hsa-miR-421	0.000000	0.250000	0.000000	0.000000
hsa-miR-29b-3p	0.000000	0.250000	0.000000	0.000000
hsa-miR-20b-5p	0.000000	0.250000	0.000000	0.000000
hsa-miR-16-5p	0.547723	-0.547723	0.547723	-0.341191
hsa-miR-146b-5p	0.250000	-0.200000	-0.464238	0.613572
hsa-miR-574-3p	-0.375000	-0.500000	0.500000	0.530330
hsa-miR-328-3p	-0.182574	0.641689	0.125000	-0.454569
hsa-let-7a-5p	0.091287	-0.456435	-0.091287	0.544062
hsa-miR-425-3p	0.000000	0.375000	0.000000	-0.303046
hsa-miR-660-5p	-0.250000	0.488094	-0.250000	0.040406
hsa-miR-223-5p	0.000000	0.000000	0.000000	0.000000
hsa-miR-362-3p	0.000000	0.000000	0.000000	0.000000
hsa-miR-132-3p	0.000000	-0.125000	0.125000	0.000000
hsa-miR-485-3p	0.000000	-0.500000	0.500000	0.000000
hsa-miR-532-5p	0.000000	0.000000	0.000000	0.000000
hsa-miR-382-5p	0.000000	0.000000	0.000000	0.000000
hsa-miR-200a-3p	0.000000	0.000000	0.000000	0.000000
hsa-miR-106a-5p	0.091287	-0.456435	-0.091287	0.414963
hsa-miR-32-5p	-0.488094	-0.125000	0.000000	0.542957

hsa-miR-133b	0.143019	-0.500000	-0.250000	0.530330
hsa-miR-152-3p	0.500000	-0.286039	0.000000	-0.303046
hsa-miR-532-3p	-0.250000	0.375000	-0.250000	0.000000
hsa-miR-451a	0.365148	-0.639010	0.182574	-0.036886
hsa-miR-23b-3p	0.365148	0.000000	-0.365148	-0.143531
hsa-miR-128-3p	-0.250000	0.375000	0.000000	-0.303046
hsa-miR-30e-5p	0.000000	-0.456435	0.238366	0.037516
hsa-miR-222-3p	0.092848	-0.371391	0.047673	0.013890
hsa-miR-339-3p	-0.250000	0.000000	0.000000	0.000000
hsa-miR-326	-0.375000	0.108465	0.300000	-0.303046
hsa-miR-497-5p	-0.300000	0.250000	-0.250000	0.000000
hsa-miR-92b-3p	0.000000	0.000000	0.000000	-0.303046
hsa-miR-652-3p	-0.600000	-0.278543	0.047673	0.527823
hsa-miR-28-5p	0.000000	-0.476731	0.125000	0.000000
hsa-miR-25-3p	0.365148	-0.547723	0.091287	-0.267420
hsa-let-7d-5p	-0.200000	-0.365148	0.182574	-0.009631
hsa-miR-1260a	0.182574	0.182574	-0.182574	-0.645497
hsa-miR-181a-5p	-0.371391	-0.550019	-0.286039	0.707910
hsa-miR-185-5p	0.091287	-0.639010	0.456435	-0.442627
hsa-miR-125a-5p	0.045835	-0.456435	0.137505	-0.267420
hsa-let-7c-5p	-0.091287	-0.639010	-0.182574	0.258199
hsa-miR-505-3p	0.000000	0.250000	-0.488094	-0.454569
hsa-miR-18a-5p	-0.108465	-0.547723	-0.185695	0.141421
hsa-miR-93-5p	-0.365148	-0.456435	0.137505	-0.018443
hsa-miR-141-3p	0.108465	-0.125000	-0.250000	-0.454569
hsa-let-7b-3p	-0.456435	0.456435	-0.381385	-0.393552
hsa-miR-486-5p	0.100000	-0.500000	0.000000	-0.444467
